# Glutathione Modulates Hydrogen Sulfide Release and the Ocular Hypotensive Action of Diallyl Polysulfide Compounds

**DOI:** 10.3390/ph17101408

**Published:** 2024-10-21

**Authors:** Susmit Mhatre, Rai Anjali, Pulkit Sahai, John Auden, Somnath Singh, Ya Fatou Njie Mbye, Sunny E. Ohia, Catherine A. Opere

**Affiliations:** 1 Department of Pharmacy Sciences, School of Pharmacy and Health Professionals, Creighton University, Omaha, NE 68178, USA or mhatre2@wisc.edu (S.M.); or rai27@wisc.edu (R.A.); pulkitsahai@creighton.edu (P.S.); johnauden@creighton.edu (J.A.); somnathsingh@creighton.edu (S.S.); 2 Cell and Molecular Biology Graduate Program, University of Wisconsin-Madison, Madison, WI 53706, USA; 3 Department of Neuroscience, University of Wisconsin-Madison, Madison, WI 53706, USA; 4 Comparative Biomedical Sciences Graduate Program, University of Wisconsin-Madison, Madison, WI 53706, USA; 5 School of Veterinary Medicine, University of Wisconsin-Madison, Madison, WI 53706, USA; 6 Department of Pharmaceutical Sciences, College of Pharmacy and Health Sciences, Texas Southern University, Houston, TX 77004, USA; yafatou.mbye@tsu.edu (Y.F.N.M.); sunny.ohia@tsu.edu (S.E.O.)

**Keywords:** hydrogen sulfide, glaucoma, intraocular pressure, glutathione, diallyl disulfide, diallyl trisulfide

## Abstract

Background: Hydrogen sulfide (H_2_S) is an endogenous transmitter with the potential to regulate aqueous humor dynamics and protect retinal neurons from degeneration. The aim of the present study was two-fold: (a) to evaluate the release of H_2_S from two polysulfides, diallyl disulfide (DADS), and diallyl trisulfide (DATS); and (b) to investigate their ocular hypotensive actions in normotensive male and female rabbits in the presence and absence of GSH. Materials and Methods: H_2_S was quantified hourly for up to 6 h using a H_2_S-Biosensor (World Precision Instruments, Sarasota, Fl). Intraocular pressure (IOP) was assessed in normotensive New Zealand Albino rabbits using a pneumotonometer (model 30 classic; Reichert Ophthalmic Instruments, Depew, NY, USA). Results: In the presence of GSH, there was an increase in the in vitro release of H_2_S produced by DADS and DATS. Both DADS and DATS also caused a dose-dependent reduction in IOP in male and female rabbits, in both treated and untreated eyes. For instance, in male animals, the presence of GSH (3% and 5%) significantly (*p* < 0.05, *n* = 5) enhanced the ocular hypotensive action of DADS (2%) and DATS (2%) from 14.02 ± 2.89% to 18.67 ± 5.6% and from 16.22 ± 3.48 to 23.62 ± 5.79%, respectively. Conclusions: GSH enhanced both H_2_S release and ocular hypotensive action of the polysulfides in a manner that was dependent on the number of sulfur atoms present in each polysulfide. Furthermore, female animals were less sensitive to the IOP-lowering action of the polysulfides, when compared to their male counterparts.

## 1. Introduction

Hydrogen sulfide (H_2_S), an inflammable toxic gas with application in organic chemistry as base material for elemental sulfur, has found applications in pharmacology after its discovery as an endogenous gaseous transmitter in various biological systems [1,2]. H_2_S has been reported to mediate multiple critical processes such as neurotransmission [2,3,4], cytoprotection [5,6,7,8], neuroprotection [6,9], smooth muscle relaxation [10,11], vasorelaxation [10,12,13,14,15,16,17], anti-inflammation [18,19,20,21,22,23], and cellular respiration [24,25], and it can serve as a free radical scavenger [2,26], amongst others.

H_2_S is endogenously produced in mammalian ocular tissues based on reports of its presence in tissues of the anterior and posterior segments [4]. Furthermore, the enzymes responsible for H_2_S biosynthesis, cystathionine-β-synthase (CBS), cystathionine-γ-lyase (CSE), and 3-mercaptopyruvate sulfurtransferase (3MST), along with cysteine aminotransferase (CAT), have been localized in different ocular tissues [4,27,28,29]. CBS insufficiency has been associated with ocular conditions such as lens dislocation, retina degeneration, retinal detachment, and acute glaucoma [30], suggesting a pivotal role for H_2_S in the pathophysiology of eye diseases. Compounds that release H_2_S in biological media such as sodium hydrosulfide (NaHS) have been reported to exert pharmacological actions in ocular tissues. For example, H_2_S-releasing compounds have been reported to relax iris smooth muscle via K^+^-ATP channel-dependent mechanisms [4], decrease the electrically evoked norepinephrine release [4] and norepinephrine levels in mammalian anterior uvea [4], and increase aqueous humor outflow facility in the anterior segment of the eye [4]. Additionally, H_2_S attenuates excitatory amino acid neurotransmission in retinal neurons [3], suggesting a neuroprotective role for this gas in the posterior segment of the eye. The ability to measure H_2_S in biological tissues involves the use of several methodologies, such as colorimetric assays for methylene blue formation, sulfide-ion-selective or -polarographic electrodes, high-performance liquid chromatography analysis with fluorescent derivatization of sulfides, and gas chromatography with flame photometric or sulfur chemiluminescence detection [31].

**Figure 1:**
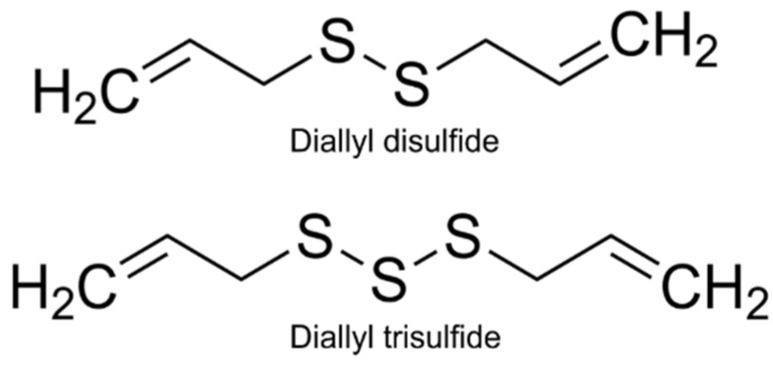
Chemical structure of diallyl disulfide (DADS) and diallyl trisulfide (DATS) with two allyl groups on each end, with either two or three sulfur atoms with disulfide bonds.

We have evidence that the inorganic compound, NaHS; a slow-releasing synthetic H_2_S donor, GYY4137; the H_2_S-latanoprost hybrid compound, ACS67; and the precursor for the endogenous production of H_2_S, L-cysteine, can reduce IOP in male New Zealand Albino normotensive rabbits [4]. However, the effect of the garlic-derived, bioactive diallyl compounds diallyl disulfide (DADS) and diallyl trisulfide (DATS) on IOP have not been described. Both DADS and DATS are bioactive compounds generated via the decomposition of allicin, the oil-soluble organosulfur derived from garlic [32]. These compounds are structurally composed of disulfide and trisulfide groups in between two allyl groups, respectively (Figure 1), and are reported to release H_2_S by nucleophilic substitution at the sulfur atom via their interaction with GSH [33]. Moreover, both DADS and DATS are reported to exhibit potent anticancer [32,34,35], cardioprotection [33], and anti-inflammatory [36] properties. Indeed, it has been postulated that production of H_2_S from the polysulfides such as DADS and DATS account for the beneficial actions of garlic [33]. In ocular tissues, we have evidence that DATS mitigated both time-dependent and oxidative stress-induced cataractogenesis via an increase in both GSH content and superoxide dismutase activity in isolated bovine lenses [37,38]. Therefore, the aim of the present study was two-fold: (a) to evaluate the in vitro release of H_2_S from DADS and DATS in the presence and absence of GSH and (b) to elucidate the ocular hypotensive action of these two diallyl compounds on IOP in the presence and absence of GSH in normotensive male and female New Zealand Albino rabbits.

## 2. Results

### 2.1. In Vitro Release

**Figure 2:**
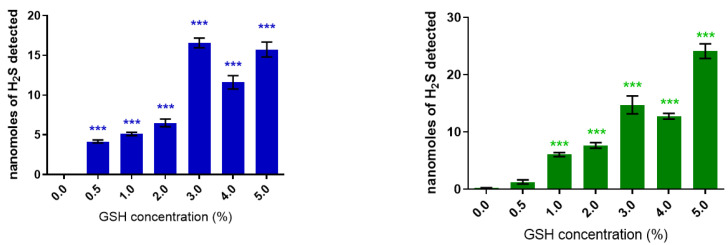
Burst release of hydrogen sulfide (H_2_S) from diallyl disulfide (DADS, **left panel**) and diallyl trisulfide (DATS, **right panel**) in the presence of glutathione (GSH), in vitro. Vertical bars represent means ± SEM; each data point represents 3 observations. One-way ANOVA *** *p* < 0.001 significantly different from control.

**Figure 3:**
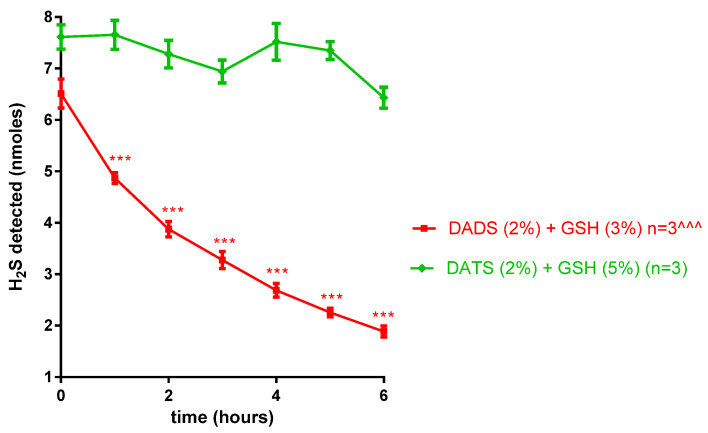
Time-dependent release of hydrogen sulfide (H_2_S) from diallyl disulfide (DADS) and diallyl trisulfide (DATS) in the presence of glutathione (GSH), in vitro. Vertical bars represent means ± SEM; each data point represents 3 observations. Two-way ANOVA ^^^ *p* < 0.001, significantly different from DATS/GSH; one-way ANOVA *** *p* < 0.0001, significantly different from H_2_S at time zero.

Whereas DADS (2%) did not release any H_2_S in solution for 6 h, DATS (2%) exhibited an immediate release of H_2_S, with a small peak of 0.2 ± 0.004 nmoles/mL observed after 5 h. The presence of GSH (0.5–5%) stimulated an instantaneous production of H_2_S from DADS (2%) that achieved a maximum release of 15.26 ± 1.29 nmoles (*p* < 0.001; *n* = 3) at the GSH (3%) concentration (Figure 2, left panel). Furthermore, DATS (2%) released H_2_S in a concentration-dependent manner, achieving a maximum release of 23.79 ± 1.03 nmoles (*p* < 0.001; *n* = 3) at the GSH (5%) concentration (Figure 2, right panel). Following the burst release, DADS (2%) in the presence of GSH (3%) exhibited a steady decline in H_2_S release in a time-dependent manner, while DATS (2%) in the presence of GSH (5%) sustained the release of H_2_S for over a period of 6 h (Figure 3).

### 2.2. Effect of Polysulfides on Intraocular Pressure

**Figure 4:**
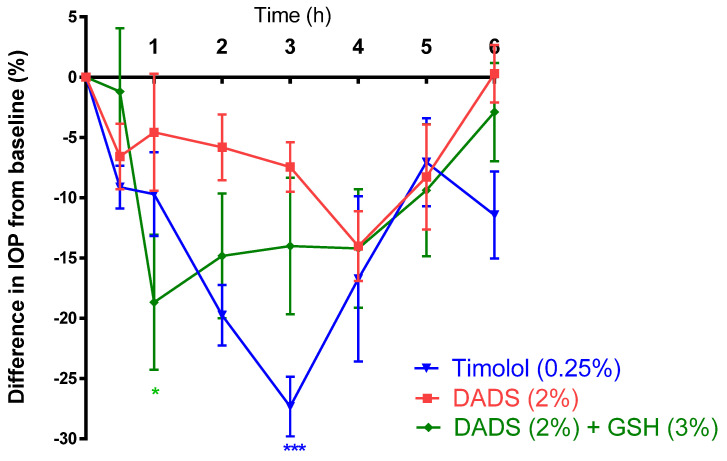
Effect of diallyl disulfide (DADS) on intraocular pressure (IOP) in the treated eye in the presence and absence of glutathione ethyl ester (GSH) in male normotensive conscious New Zealand Albino rabbits in vivo. Vertical bars represent means ± SEM of data obtained from five rabbits. * *p* < 0.05; *** *p* < 0.001, significantly different from baseline IOP.

Both of the diallyl polysulfides, DADS and DATS, exerted an ocular hypotensive effect on male New Zealand Albino rabbits. DADS (2%) achieved a maximum (*p* > 0.05; *n* = 5) reduction in IOP of 14.02 ± 2.89% after 4 h in the treated eye, and 6.51 ± 1.45% after 30 min in the contralateral eye (Figure 4 and Table 1). Whereas glutathione (GSH) did not significantly alter IOP pretreatment of animals with GSH (3%) significantly (*p* < 0.05, *n* = 5) enhanced the maximum IOP-lowering action of DADS (2%) to 18.67 ± 5.60% (Figure 4) after 1 h in the treated eye, while the contralateral eye exhibited a reduction of 15.69 ± 2.24% in 1 h as well (Table 1). Whereas topical administration of the vehicle (DMSO (20%)/saline) and normal saline, which served as negative controls, did not elicit a significant effect on IOP in all normotensive New Zealand Albino rabbits, the IOP-lowering action of DADS (2%) was less than that of timolol (0.25%; used as a positive control), which elicited a maximal effect of 27.33 ± 2.46% (*p* < 0.001; *n* = 5) in 3 h in the treated eye and 18.86 ± 6.59% (*p* > 0.05; *n* = 5) (Figure 4).

**Figure 5:**
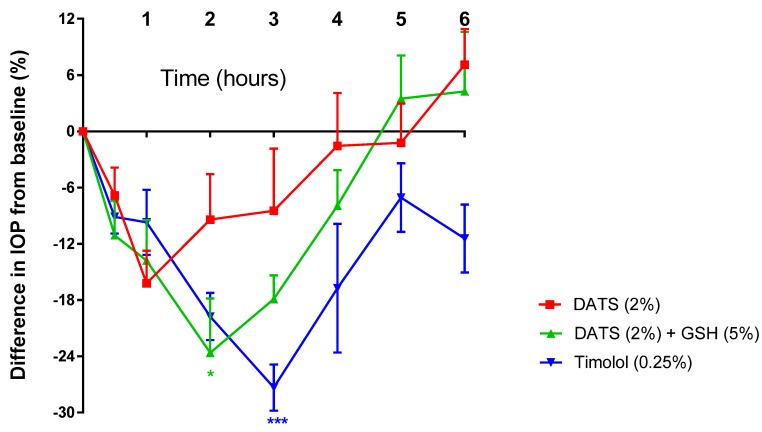
Effect of diallyl trisulfide (DATS) on intraocular pressure (IOP) in the treated eye in the presence and absence of glutathione ethyl ester (GSH) in male, normotensive conscious New Zealand Albino rabbits in vivo. Vertical bars represent means ± SEM of data obtained from five rabbits. * *p* < 0.05; *** *p* < 0.001, significantly different from baseline IOP.

It was interesting to note that DATS (0.1%, 1%, and 2%) elicited a reduction in IOP in a concentration-dependent manner. For instance, in male animals, DATS (0.1%; 1% and 2%) achieved a maximum reduction in IOP of 11.03 ± 2.57%, 13.08 ± 2.01%, and 16.22 ± 3.48 (*p* > 0.05; *n* = 5) in 0.5, 2, and 1 h, respectively, in the treated eye (Table 2 and Figure 5). In the contralateral eye, DATS (0.1%; 1% and 2%) achieved a maximum reduction in IOP by 11.70 ± 4.85%, 8.59 ± 6.78, and 9.38 ± 4.17% (*p* > 0.05; *n* = 5) in 1, 5, and 1 h, respectively (Table 2).

The presence of GSH-EE (5%) enhanced the IOP-lowering effect of DATS (2%), achieving a significant (*p* < 0.05; *n* = 5) maximum reduction of 23.62 ± 5.79% and 21.14 ± 7.42% in the treated and contralateral eyes in 2 h, respectively (Figure 5 and Table 2). It was interesting to note that this decrease in the IOP was either less than (for the treated eye) or comparable (for the contralateral eye) to that elicited by timolol (0.25%) in male animals (Figure 5 and Table 2).

**Figure 6:**
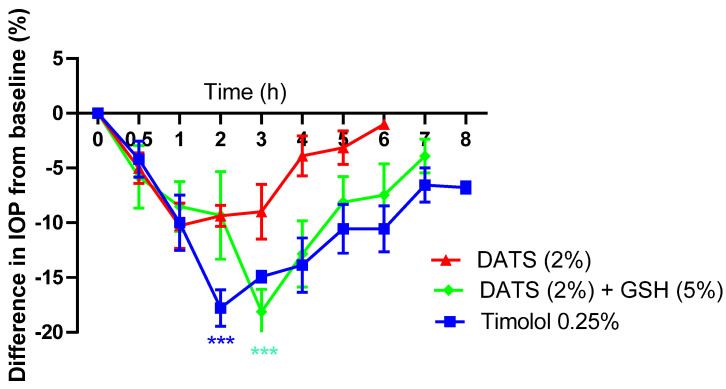
Effect of diallyl trisulfide (DATS) on intraocular pressure (IOP) in the treated eye in the presence and absence of glutathione ethyl ester (GSH) in female normotensive conscious New Zealand Albino rabbits in vivo. Vertical bars represent means ± SEM of data obtained from five rabbits. *** *p* < 0.001, significantly different from baseline IOP.

To determine the role of gender in the IOP-lowering effect of polysulfides, the effect of DATS was evaluated in female normotensive animals. DATS (2%) elicited a lower IOP-lowering action in females (10.26 ± 2.06%; *p* > 0.05), compared to 16.22 ± 3.48% (*p* > 0.05) in males after 1 h in the treated eye. The contralateral effect for DATS (2%) was insignificant and equivalent in males (9.38 ± 4.17%) and females (8.05 ± 3.29 (Figure 6 and Table 3a,b). The presence of GSH (5%) enhanced the maximum IOP-lowering action of DATS (2%) to 18.14 ± 2.08% (*p* < 0.01; *n* = 5) in 3 h in females, compared to 23.62 ± 5.79 (*p* < 0.05) in 2 h in males in the treated eye (Figure 6 and Table 3a,b). The contralateral effect was higher in males (21.14 ± 7.42%; *p* < 0.05 in 3 h) compared to that in females (11.14 ± 2.92%; *p* > 0.05 in 4 h). Moreover, timolol elicited a lower ocular hypotensive effect in females as well (17.78 ± 1.68; *p* > 0.05 in 2 h) compared to that in males (27.33 ± 2.46; *p* < 0.001 in 3 h). The contralateral effect of timolol was not significant in both males and females (Figure 6 and Table 3a,b).

## 3. Discussion

In the past few decades, H_2_S has evolved from an industrial toxicant to a gaseous transmitter that plays significant pathophysiological roles in multiple biological systems [39]. The potential therapeutic applications of this gas have been described for several diseases, including cardiovascular, inflammation, cancer, Parkinson’s disease, Alzheimer’s disease, etc. [39,40]. In ocular tissues, several investigators have localized the enzymes responsible for the biosynthesis of H_2_S in the retina, iris ciliary bodies, lens, etc. [4,27,28,29]. In the anterior segment of the eye, H_2_S modulates sympathetic neurotransmission [4] and relaxes smooth muscle [4] in mammalian isolated iris ciliary bodies in vitro. In the posterior segment, H_2_S-releasing compounds have also been reported to relax posterior ciliary arteries and to inhibit amino acid neurotransmission in the neural retina [3,4]. We have further demonstrated that multiple H_2_S-producing compounds, NaHS, GYY4137, and ACS67, can reduce IOP in normotensive male New Zealand Albino rabbits [4]. However, the effect of the diallyl compounds DADS and DATS in ocular tissues has not been investigated. DADS and DATS are bioactive polysulfide compounds generated from the decomposition of allicin and are believed to contribute to beneficial roles of garlic via the production of H_2_S. Therefore, this study investigated the in vitro release of H_2_S from DADS and DATS and their ocular hypotensive action in normotensive male and female New Zealand Albino rabbits.

It was interesting to note that the DADS did not release any detectable H_2_S in aqueous solution, while DATS exhibited a small release of the gas over a period of 6 h. The cellular release of H_2_S from the polysulfides is reported to be dependent on the presence of GSH. Therefore, in vitro, the release of H_2_S was further determined in the presence of GSH, where the presence of exogenous GSH (0.5 to 5%) triggered a burst release of the H_2_S from both polysulfides. The maximum burst production of H_2_S from DADS was observed after GSH (3%), while that of DATS was concentration-dependent, achieving a maximum production at the highest concentration tested, GSH (5%). Moreover, DATS exhibited a higher and consistent rate of H_2_S release compared to that of DATS for the 6-h duration of the study. In corroboration, DADS has been reported to exhibit less burst release and a slower rate of release compared to that of DATS in PBS [39,41]. Benavides et al. (2007) proposed that both DATS and DADS release H_2_S via an α-carbon nucleophilic substitution pathway which requires an interaction with GSH [33]. However, Liang et al. (2015) contended that DADS does not significantly produce H_2_S via the α-carbon nucleophilic reaction with GSH but rather interacts with GSH via thiol–disulfide exchange to form products that do not release H_2_S. These researchers further proposed that the presence of DATS impurities in commercially available DADS could account for the H_2_S production observed from DADS [41]. It also is conceivable that, irrespective of mechanism, DADS, which contains two sulfur atoms, can potentially produce less H_2_S compared to DATS, which has three sulfur atoms and can produce more H_2_S per molecule of each polysulfide. In preliminary in vitro studies, we observed that exogenous glutathione (0.5–5%) released a small, insignificant amount of H_2_S in solution. Furthermore, these concentrations of GSH did not affect IOP but enhanced the maximum IOP-lowering actions of the polysulfides. Since the purpose of this study was to determine the effect of GSH-mediated H_2_S release on IOP, subsequent in vivo experiments were conducted using GSH quantities that exhibited the highest H_2_S release for each polysulfide (3% GSH for DADS and 5% for DATS).

To determine if the release of H_2_S from the polysulfides correlates with their IOP-lowering ability, the effect of topically administered DADS and DATS was determined in normotensive male and female New Zealand Albino rabbits. GSH has been localized in mammalian ocular tissues such as the lens, cornea, retina, aqueous humor, etc., where it plays an integral role in the maintenance of ocular homeostasis and defensive protective mechanisms against oxidative stress in ocular tissues [42,43]. Therefore, endogenous GSH was leveraged to trigger the in vivo release of H_2_S via undergo nucleophilic substitution reaction. Although DADS (2%) did not produce any detectable H_2_S, it exhibited a minor reduction in IOP (14.02 ± 2.89%; *p* > 0.05; *n* = 5) that could be attributed to the presence of endogenous GSH in the anterior uvea of the eye. Interestingly, DATS (2%), which released a small quantity of H_2_S in vitro, also elicited a minor reduction in IOP (16.22 ± 3.48 (*p* > 0.05; *n* = 5). Taken together, these observations suggest that endogenous GSH does not trigger H_2_S production from the polysulfides in quantities sufficient to significantly reduce IOP. However, the magnitude of IOP reduction appears to be proportional to the number of sulfur atoms in each polysulfide.

Congruent to the in vitro H_2_S-release studies, the presence of exogenous GSH enhanced the IOP-lowering action of both polysulfides in the treated and contralateral eyes of male normotensive animals. In addition to the production of H_2_S from DATS via the α-carbon nucleophilic substitution pathway [33], in mammalian cells, DATS has been proposed to generate endogenous H_2_S via other mechanisms, including interaction with other thiol–SH proteins, upregulation of the biosynthetic enzyme CSE, and via an oxidoreductase reaction with catalase enzyme [39]. Indeed, these mechanisms may contribute to the higher rate of H_2_S production from DATS. It is conceivable that, in the presence of GSH, H_2_S produced in the anterior chamber easily diffuses through cell membranes to gain central access and distributes to the contralateral eye [44], where it elicits its hypotensive effects. Consistent with the in vitro release studies, the IOP-lowering effect of DATS/GSH was higher than that of DADS/GSH in both the treated and contralateral eyes. In corroboration, Kim et al. (2014) reported that the antioxidant capacity of the polysulfides to activate nuclear factor erythroid 2-related factor 2 (Nrf2), a transcription factor that regulates the cellular defense against toxic and oxidative insults, in human gastric epithelial cells was directly proportional to the number of sulfur atoms in each polysulfide (DATS > DADS > DAS) [45]. Taken together, IOP reduction correlates with H_2_S production, both in the absence and presence of GSH.

To determine the role of gender in the IOP-lowering action of the polysulfides, the effect of DATS (2%) in males was compared to that in females in normotensive animals. In general, female animals were more resistant to the IOP-lowering action of DATS and timolol when compared to male animals. To the best of our knowledge, no gender-related responses to the IOP-lowering action of H_2_S-releasing compounds have been reported in the literature for rabbits. Wang et al. (2013) conducted studies to assess IOP over a 24-h period in normotensive New Zealand rabbits and found no significant gender differences in IOP between males and females [46]. However, exogenous progesterone was associated with an increase in IOP in normotensive rabbits, implying that elevation in progesterone associated with the luteal phase of the reproductive cycle could disrupt IOP [47,48]. Since the reproductive cycle in the female animals in this study was unknown, it is conceivable that variations associated with the reproductive cycle could contribute to the apparent resistance to the IOP-lowering actions of the drugs tested.

## 4. Materials and Methods

### 4.1. In Vitro Hydrogen Sulfide Release Studies

To quantify the release of H_2_S from DADS and DATS, stock solutions (25 mg/250 µL) were prepared in DMSO and GSH (0.5–5%) in argonated/deionized water. Aliquots of GSH (0.5–5%) were added to each diallyl compound (2%; 10 µL), and the volume was stocked up to 100 µL using argonated water, as indicated on Table 4. The amount of H_2_S was quantified amperometrically using a H_2_S-Biosensor ion-selective electrode and TBR4100 Free Radical Analyzer, both from World Precision Instruments (WPI), (Sarasota, FL, USA), using a protocol described elsewhere [49].

### 4.2. Intraocular Pressure Studies in Normotensive Rabbits

Animal protocols were approved by the Institutional Animal Care and Use Committee (Protocol #1165, 2021). Animal studies were conducted in adherence to the Association for Research in Vision and Ophthalmology’s statement for the Use of Animals in Ophthalmic and Vision Research.

Male and female normotensive New Zealand Albino rabbits (weighing about 2 kg) were purchased from Charles River Laboratories, Wilmington, MA, USA. In addition to conditioning to 12-h light–dark cycles, animals were acclimatized for 7 days and divided into groups of five animals/group (n = 5). On the day of the experiment, two baseline IOP measurements were taken following topical application of proparacaine 0.5% (local anesthetic) and averaged to serve as the baseline reading for comparison to IOP readings following the treatment. IOP readings were taken using a Pneumatonometer (model 30 classic; Reichert Ophthalmic Instruments, Depew, NY, USA). At 0 h, 50 µL of the test compounds (DATS and DATS) was applied topically to one eye of each animal, while the contralateral eye received the same quantity of vehicle (saline). IOP readings were obtained at 30 min, 1 h, and 2 h and then every hour until the IOP returned to baseline. When used, GSH-EE was topically administered 10 min prior to the topical application of H_2_S-producing compounds. GSH-EE was used in the in vivo studies instead of reduced GSH, which was used in in vitro studies to increase access of the intracellular antioxidant into the anterior chamber of the eye. Normal saline and vehicle (DMSO/saline/Tween 20) were used as negative controls, while timolol was used as a positive control. Animal eyes were monitored for signs of ocular discomfort, such as tearing, redness, conjunctival swelling, squinting, and eyelid irritation, that could occur due to the administration of experimental compounds. At the conclusion of the study, animals were painlessly euthanized, as approved by IACUC, using intravenous injection of Euthanasia solution (1 mL/10 lb weight; 1 mL contains 390 mg pentobarbital sodium and 50 mg phenytoin sodium, Patterson Veterinary, Patterson Vet Supply, Inc., manufactured for: Virbac AH, Inc, Fort Worth, TX, USA).

### 4.3. Data Analysis

Results were expressed as change in IOP (mm of Hg) and/or percentage inhibition of IOP. Except where indicated otherwise, values given are arithmetic means ± S.E.M. Significance of differences between control and agent-treated preparations was evaluated using analysis of variance (ANOVA), followed by Tukey’s post-test. Differences with *p*-values < 0.05 were accepted as statistically significant.

## 5. Conclusions

**Figure 7:**
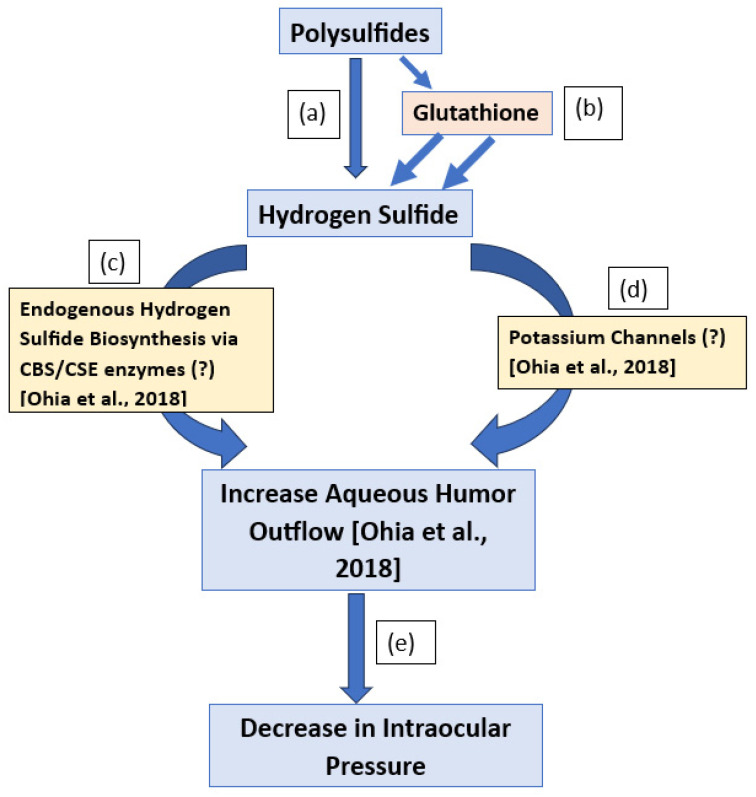
Schematic presentation of the of the possible pharmacological mechanisms by which polysulfides decrease intraocular pressure in normotensive male and female New Zealand Albino rabbits. The production of hydrogen sulfide from polysulfides (**a**) is enhanced in the presence of glutathione (**b**). Hydrogen sulfide elicits an increase in aqueous humor outflow (**e**) via CBS/CSE biosynthetic pathways (**c**) and/or potassium channels (**d**) to reduce intraocular pressure (**e**) [4].

Although DADS did not release H_2_S in solution, while DATS released minute quantities of H_2_S in a simple aqueous environment, both polysulfides elicited a burst release of the gas in the presence of GSH. The presence of GSH elicited an increased release of the gas from DATS, compared to DADS. The IOP-lowering action of the polysulfides was directly dependent on the number of sulfur atoms in each polysulfide and was enhanced by the presence of GSH. Moreover, female animals were less sensitive to the IOP-lowering effects of the polysulfide DATS. The possible pharmacological mechanisms by which the polysulfides decrease IOP in normotensive male and female New Zealand Albino rabbits are shown in Figure 7.

## Figures and Tables

**Table 1:** Effect of DADS on IOP in the contralateral eyes of normotensive male New Zealand Albino rabbits.

Treatment	Contralateral Eye		
	Peak Activity (h)	Duration of Action (h)	Maximum % IOP Reduction Mean ± SEM
DADS (2%)	0.5	2	6.51 ± 1.45
DADS (2%) + GSH (3%)	1	6	15.69 ± 2.24
Timolol gel drops (0.25%)	3	6	18.86 ± 6.59

Effects of DADS (2%) in the absence and presence of GSH (3%) were not significantly different from baseline IOP (*p* > 0.05). Similarly, the effect of timolol, the positive control, was not statistically significant from baseline IOP (*p* > 0.05). Each IOP reading represent means ± SEM of data obtained from five rabbits. DADS, diallyl disulfide; GSH, glutathione ethyl ester; IOP, intraocular pressure.

**Table 2:** Effect of DATS on IOP in the treated eye normotensive male New Zealand Albino rabbits.

Treatment	Treated Eye			Contralateral Eye		
	Peak Activity (h)	Duration of Action (h)	Maximum % IOP Reduction Mean ± SEM	Peak Activity (h)	Duration of Action (h)	Maximum % IOP Reduction Mean ± SEM
DATS (0.1%)	0.5	2	11.03 ± 2.57	1	4	11.70 ± 4.85
DATS (1%)	2	8	13.08 ± 2.01	5	8	8.59 ± 6.78
DATS (2%)	1	6	16.22 ± 3.48	1	6	9.38 ± 4.17
DATS (2%) + GSH (5%)	2	5	23.62 ± 5.79 *	3	6	21.14 ± 7.42 *
Timolol (0.25%)	3	6	27.33 ± 2.46 ***	3	6	18.86 ± 6.59

Each IOP reading represent means ± SEM of data obtained from five rabbits. * *p* < 0.05 means that the effect of DATS (2%) in the presence of GSH (5%) was significantly different from baseline IOP in the treated and the contralateral eye. *** *p* > 0.001 means that the effect of timolol was significantly different from baseline IOP in the treated eye. DATS, diallyl trisulfide; GSH, glutathione; IOP, intraocular pressure.

**Table 3:** (**a**) Effect of DATS on IOP in the treated eye in normotensive male and female New Zealand Albino rabbits. (**b**) Effect of DATS on IOP in the contralateral eye in normotensive male and female New Zealand Albino rabbits.

a						
Treatment	Male, Treated Eye			Female, Treated Eye		
	Peak Activity (h)	Duration of Action (h)	Maximum % IOP Reduction Mean ± SEM	Peak Activity (h)	Duration of Action (h)	Maximum % IOP Reduction Mean ± SEM
DATS (2%)	1	6	16.22 ± 3.48	1	6	10.26 ± 2.06
DATS (2%) + GSH (5%)	2	5	23.62 ± 5.79 *	3	7	18.14 ± 2.08 **
Timolol (0.25%)	3	6	27.33 ± 2.46 ***	2	8	17.78 ± 1.68
**b**						
**Treatment**	**Male, Contralateral Eye**			**Female, Contralateral Eye**		
	**Peak Activity (h)**	**Duration of Action (h)**	**Maximum % IOP Reduction Mean ± SEM**	**Peak Activity (h)**	**Duration of Action (h)**	**Maximum % IOP Reduction Mean ± SEM**
DATS (2%)	1	6	9.38 ± 4.17	3	6	8.05 ± 3.29
DATS (2%) + GSH (5%)	3	6	21.14 ± 7.42 *	4	7	11.14 ± 2.92
Timolol (0.25%)	3	6	18.86 ± 6.59	2	8	13.17 ± 2%

Each IOP reading represent means ± SEM of data obtained from five rabbits. (a) * *p* < 0.05; ** *p* < 0.01 mean that the effect of DATS (2%) in the presence of GSH (5%) was significantly different from baseline IOP in the treated eyes. *** *p* > 0.001 means that the effect of timolol was significantly different from baseline IOP in the treated eye. (b) * *p* < 0.05 means that the effect of DATS (2%) in the presence of GSH (5%) was significantly different from baseline IOP in the contralateral, untreated eye. DATS, diallyl trisulfide; GSH, glutathione; IOP, intraocular pressure.

**Table 4:** Preparations of diallyl compounds with GSH for assessment of in vitro release of H_2_S.

Target GSH %	10% Donor Solution (µL)	GSH 5% (µL)	Argonated Water (µL)	Total (µL)
0%	10	0	90	100
0.5%	10	5	85	100
1%	10	10	80	100
2%	10	20	70	100
3%	10	30	60	100
4%	10	40	50	100
5%	10	50	40	100

## Data Availability

The original contributions presented in the study are included in the article, further inquiries can be directed to the corresponding author.
